# Effect of Different Dietary Betaine Fortifications on Performance, Carcass Traits, Meat Quality, Blood Biochemistry, and Hematology of Broilers Exposed to Various Temperature Patterns

**DOI:** 10.3390/ani11061555

**Published:** 2021-05-26

**Authors:** Ahmed A. Al-Sagan, Abdullah H. Al-Yemni, Alaeldein M. Abudabos, Abdulaziz A. Al-Abdullatif, Elsayed O. Hussein

**Affiliations:** 1 King Abdulaziz City for Science & Technology, P.O. Box 6086, Riyadh 11442, Saudi Arabia; 2 Arabian Agriculture Services Company (ARASCO), Riyadh 11593, Saudi Arabia; abdullah@arasco.com; 3 Department of Animal Production, College of Food and Agriculture Sciences, King Saud University, Riyadh 11451, Saudi Arabia; aabudabos@ksu.edu.sa (A.M.A.); aalabdullatif@ksu.edu.sa (A.A.A.-A.); shessin@ksu.edu.sa (E.O.H.)

**Keywords:** broilers, heat stress, betaine, performance, meat quality, blood constituents

## Abstract

**Simple Summary:**

Enhancing production of broilers in hot climates is essential to overcoming high-heat problems. High temperature during the summer season is one of the most influential factors affecting poultry production. Heat stress has long been identified as dampening growth performance and increasing vulnerability to diseases. Several methods have been used to alleviate the adverse effects of heat stress. One of these is the dietary fortification of betaine. Betaine (Trimethylglycine) naturally occurs in various plants, and it can be extracted from beetroot. The current experiment examined the effects of dietary betaine fortification on broiler chickens at 1–40 days of age. At 18 days of age, half of the birds were kept under thermos-neutral temperature (22–24 °C), while the other half were kept under high temperature (35 °C). The production efficiency factor was best (*p* < 0.05) for birds that received 0.10% betaine. Betaine fortification improved the growth, feed utilization, and production index. Betaine fortification at 0.1% betaine supplementation with an 84 g increase in growth, 4.6 points improvement in feed utilization, and 24 points enhancement in production index compared with no betaine fortification. Betaine fortification (0.1%) during heat stress reduced the negative impact on performance and improved production efficiency, suggesting that betaine may be as a practical tool for improving poultry production in hot regions.

**Abstract:**

Improving broilers’ production in the hot region is essential to overcome heat-stress challenges. The current experiment examined the effects of betaine’s fortification (0.0, 0.075, 0.10, and 0.15%) to broiler chickens during days 1–40 of age. The growth period was divided into the starter (1–18 d) and growing-finishing (19–40 d). During the starter period, there was no heat challenge, and all birds were kept under the same conditions. At 18 days of age, half of the birds were kept under thermos-neutral temperature (TN, 22–24 °C), while the other half were kept under high temperature (HT, 35 °C). However, the production efficiency factor (PEF) was the best (*p* < 0.05) for birds that received 0.10% betaine. Betaine fortification improved (*p* < 0.05 and 0.01) body weight gain (BWG), feed conversion ratio (FCR), and production efficiency factor (PEF) in the cumulative finisher heat-stress challenge period (19–40 d). The best performance was achieved at 0.1% betaine fortification with 84 g gain, 4.6 points improvement in FCR, and 24 points improvements in PEF as compared to no betaine fortification. The heat-stressed group consumed less feed (239 g), gained less weight (179 g), converted feed less efficiently (2.6 points), and, as a result, had lower FEF (29 points) as compared to the TN group. Conclusively, heat challenge had a powerful effect on growth performance, meat characteristics, and blood parameters, especially during the grower-finisher period. Betaine fortification (0.1%) during heat stress reduced the negative impact on performance and improved production efficiency, suggesting that betaine is a useful nutritional tool under stress conditions that deserves further investigation.

## 1. Introduction

Recently, poultry production’s husbandry and feeding protocols have become crucial issues for farmers, particularly in hot areas, as they can lessen the comparative heat burden [1,2,3,4,5]. High temperature during the summer season is one of the most significant factors affecting poultry production [6,7,8]. Heat stress has long been identified as dampening growth performance and egg production and increasing vulnerability to diseases [4,9,10,11].

Given the substantial importance of poultry products and high-quality and affordable animal protein to consumers, there is an urgency to study the factors that hinder poultry production. One of the major factors that influence poultry production is heat stress, particularly in desert regions with scorching climates, such as Saudi Arabia, which has an average annual temperature of about 43.4 °C.

Several approaches have been used to alleviate the adverse effects of heat stress [2,3,4,5,6,7,8,10]. One of these is the dietary fortification of betaine [12,13,14,15,16]. Betaine (Trimethyl glycine) naturally occurs in various plants, and it can be extracted from beetroot [12]. It is a key source of the methyl group (CH_3_) in poultry additives [1,17], and it is created by choline and glycine in cell mitochondria [12,16]. Betaine plays two valuable roles in the metabolism of the animals [18,19]. Its initial role is to provide methyl activists, and its other role is linked to its zwitterionic feature of Osmo protectant or osmolyte, which aids in preserving cell water homeostasis [20,21] without influencing cell metabolism. Osmolytes, which act as chaperones, stop or postpone stress-induced denaturation and aid previously denatured proteins [22,23]. Additionally, the methyl group is fundamental in the metabolism of poultry species, as they cannot create the methyl group and therefore require it in their diets [18,24]. Betaine is known to amplify patient sensitivity to hypertonic heat stress and reduce the demand for inducible heat-shock protein expression [17,23]. The recommended dose of betaine for broiler chickens is disputed in the literature and requires further verification, particularly under normal and heat-stress conditions. Therefore, this study aimed to determine the effect of different dietary betaine fortifications on performance, carcass traits, meat quality, blood biochemistry, and hematology of broilers exposed to various temperature patterns after 18 days of age.

## 2. Materials and Methods

### 2.1. Study Ethics and Area

The research was conducted according to the ethics and guidelines of the King Abdulaziz City for Science and Technology under project no. LGP—35-269 at the Research Station of the King Abdulaziz City for Science and Technology, Al-Muzahmiyya, which is about 50 km away from Riyadh City, Suadi Arabia.

### 2.2. Birds and Experimental Design

During the starter period, a total of 480 1-day-old broiler chicks (Ross 308) were allotted to 96 cages (60 W × 50 D × 40 H cm), with five animals per cage. Isocaloric and isonitrogenous starter (1–18 d) and grower-finisher (19–40 d) diets, in mashed form, were formulated according to the Ross 308 recommendation guide (Table 1). During the starter period (1–18 d), the animals were divided into four experimental groups, with each consisting of 24 replicates of five chicks each: the control group feed was a supplemented basal diet, while the other three groups received increasing levels of betaine (0.075%, 0.10%, and 0.15%). Betaine is an anhydrous betaine (96%), and it is a product of Selko feed additives, which is a part of Trouw Nutrition, belonging to Nutreco Company, Rotterdam, the Netherlands. Selko’s recommended dose of betaine is 1–2 g/kg for complete feed.

At 18 days of age and until the slaughter age (40 d), birds were placed in two different rooms with 48 cages per room and 12 cages per group in each room. One room was set as thermoneutral (TN), and the other was that of high-temperature (HT) treatment. For the first week, both rooms were on a 24 h light schedule. Then, they were maintained on a light–dark cycle of 20 h: 4 h. An HT cycle was applied in the HT room. The daily temperature of the HT room fluctuated between 24 and 35 °C. However, the TN room temperature was kept at 24 °C during the remaining part of the tested period. The HT chicks were exposed to 35 °C between 8:00 to 15:00. Then, the temperature was gradually decreased to 24 °C. The average outdoor temperature and relative humidity during the experimental period were 35 ± 4 and 31 ± 5, respectively.

Thus, during the growing period, the experimental groups of 12 replicates of five birds each received one of the following eight treatments as 4 betaine levels x 2 temperatures factorial arrangement in a completely randomized design. The treatments were as follows: control diet in the TN room, control diet in the HT room, control + 0.075% betaine in the TN room, control + 0.075% betaine in the HT room, control + 0.10% betaine in the TN room, control + 0.10% betaine in the HT room, control + 0.15% betaine in the TN room, and control + 0.15% betaine in the HT room.

### 2.3. Measurements

#### 2.3.1. Performance Measurements

Body weight gain (BWG) and feed intake (FI) per cage/replicate were recorded every 6 days, while the feed conversion ratio was adjusted in accordance with mortality and computed using the feed intake and BWG of each cage. The production efficiency factor (PEF) was calculated weekly by using the following formula: PEF = ((Livability × Live body weight (kg))/((Age in days × FCR)) × 100.

#### 2.3.2. Carcass and Meat Characteristic Measurements

At day 40, 12 birds were randomly selected per treatment to represent all treatment replicates (a total 96 birds) for measuring carcass characteristics and meat quality traits after being fasted overnight. After euthanasia, the jugular vein was cut, and the heads, feathers, nicks, and shanks were cut. Then, the left parts of the carcasses were cut into two quarters of leg and breast, and they were eventually weighed. The yield percentage of each section was obtained on a dressed weight basis. For the small-intestine measurements, the entire empty gastrointestinal tract was removed aseptically, the ceca and small intestine were firstly weighed, and their total length was determined. After that, the small intestine was separated into the jejunum, ileum, and duodenum, and their lengths and weights were determined. The intestine percentage was determined as the ratio between the total intestinal weight (g) and dressed weight (g).

The right and left breasts from each bird were used to determine meat quality. The breast muscle’s pH was measured at 10 min and 24 h postmortem by a microprocessor pH-meter (Model pH 211; Hanna Instruments, Woonsocket, RI, USA). The breast muscle temperature was measured at 15 min postmortem, using a portable digital thermocouple (EcoScan Temp JKT; Thermo Scientific, Waltham, MA, USA). Using a chroma meter (CR-400; Konica Minolta, Tokyo, Japan), the L* (lightness), a* (redness), and b* (yellowness) classes for the breast muscles were determined at 15 min and 24 h after slaughtering fin accordance with the Commission International de l’Eclairage (1976) [25]. The breasts were later stored at −80 °C, till analysis, to determine broilers’ meat quality traits. The frozen muscles were thawed overnight, at 4 °C, before the examination. Then the myofibril fragmentation index (MFI) of the breast muscle was determined [26], as well as the water-holding capacity (WHC) [27] and its modification [28]. Cooking loss (CL) was measured [29]. Cooked meat samples were then used to evaluate shear force (SF) by following Wheeler et al. [30].

Blood samples were taken via brachial venipuncture from 12 randomly chosen chicks from each treatment and placed into plain tubes for analysis at 35 d of age. The samples were then centrifuged at 5 °C and 3000 rpm for 10 min, and the serum was collected and stored at −80 °C until further analysis. Albumin, protein, globulin, triglycerides (TGs), cholesterol, and glucose analysis was conducted by using enzymatic colorimetric kits that were bought from Bio-diagnostic, Cairo, Egypt. The globulin concentration was recorded as the difference between albumin and protein concentrations.

Hematological parameters were assessed by following Attia and Hassan [2] and Attia et al. [3]. One hundred leukocytes were counted per slide for each bird. The ratio of heterophile to lymphocyte was determined by using differential counts between heterophils and lymphocytes and the respective percentages of each cell.

### 2.4. Statistical Analysis

Data for the starter period were analyzed by using analysis of variance (ANOVA) in a completely randomized design, using the general linear model (GLM) procedure in Statistical Analysis System (SAS) software [31]. When the means were significantly different t *p* < 0.05, Tukey’s test was applied to separate means. For the finisher period, all data were analyzed by using the SAS [31] for a 2 × 4 factorial experiment. The replicate was the experimental unit. Percentage data were log10 transformed before conducting ANOVA. Before running the statistical analyses, the normality of both the error and data distributions were tested by using the Shapiro–Wilk test of normality in SAS^®^ [31]. The ANOVA assumptions were validated according to the random selection of the samples and normal error distribution, and the data distribution and variance homogeneity.

## 3. Results

### 3.1. Experimental Diets, and Performance during the Starter Period

The basal experimental diets fed during the starter and finisher periods are displayed in Table 1. The diets were isocaloric and iso nitrogenous and supplemented with different betaine levels 0, 0.075, 0.10 and 0.15%. The diets meet the nutritional requirements of broiler chickens, depending on the starter and finisher periods.

The cumulative FI, BWG, FCR, and PEF of Ross 308 for the starter period are shown in Table 2. No significant differences in FI, BWG, and FCR were found for the cumulative period. However, the PEF was highest (*p* < 0.05) for birds that had received 0.10% betaine compared with their counterparts. The result indicated a little benefit of supplementing the chicken diets with betaine during the starter period when there was no heat stress.

### 3.2. Performance during the Grower-Finisher Period

Performance data for the grower-finisher period (19–40 d) are presented in Table 3. Betaine fortification caused significant differences (*p* < 0.05 and 0.01) in BWG, FCR, and PEF. Birds that received 0.10% betaine gained 84 g of body weight, and their FCR and PEF improved by 4.6 and 24 points, respectively, compared with birds without betaine fortification. The temperature had an evident effect on all performance parameters over the cumulative grower-finisher period. Broilers in the TN treatment consumed more feed (239 g), gained more weight (179 g), converted feed more efficiently (2.6 points), and showed higher FEF (29 points) than those in the HT treatment (Table 3). However, the interaction effect between betaine and temperature was insignificant on the growth performance indices.

### 3.3. Body Parts and Meat Characteristics

The mean percentage yield of carcass parts in different treatments at 40 d of age is documented in Table 4. The betaine level did not cause any differences in the percentage yield of carcass parts. However, temperature affected the breast yield (*p* < 0.01) and fat percentage (*p* < 0.05). The broilers that were exposed to TN had higher breast yields and lower fat percentage than the HT group. A two-way interaction (Betaine x Temperature) was significant (*p* < 0.05) for the relative intestine weight (IRW). For the TN treatment, the 0.10% betaine level significantly decreased the IRW compared with the unsupplemented TN group. Under HT conditions, betaine fortifications did not affect IRW. In the 0.10% betaine and HT treatment, the IRW was significantly higher than TN treatment.

### 3.4. Breast Quality Characteristics

Betaine did not affect the pH value when measured 10 min postmortem (Table 5). However, the temperature did: chicken breasts from the TN group had a higher pH (6.57; *p* < 0.01) than those from the HT group (6.49). Breast temperature was altered (*p* < 0.001) by the betaine level, temperature, and their interaction. Higher temperatures were obtained from the breast meat of HT-exposed broilers fed 0.10% and 0.15% betaine, while the lowest temperature was found for the unsupplemented HT group.

Yellowness (b*) and lightness (L*) values of the breast changed (*p* < 0.05) with temperature 10 min postmortem (Table 5). Higher (b*) and (L*) values were obtained for the TN group than the HT group. The values of a* (redness) were impacted (*p* < 0.05) by the betaine x temperature interaction. The results showed that the redness of meat was higher for the breast of HT-exposed broilers fed 0.15% betaine, while the lowest redness score was found for the unsupplemented, 0.15% betaine-supplemented TN group, and 0.075 betaine-supplemented HT group. The (L*) score was higher (*p* < 0.05) for the TN group than the HT group 24 h postmortem (Table 5).

Betaine did not affect the pH value when measured at 24 h postmortem (Table 5). Breasts from the TN group had a higher pH (6.39) than the HT group (6.36). The pH value at 24 h was influenced (*p* < 0.01) by the betaine x temperature interaction. After 24 h, the breast pH was higher for NS-exposed broilers fed an unsupplemented diet than most of the experimental groups, except for the TN group fed a 0.010% betaine-supplemented diet. Betaine supplementation at 0.10% decreased the breast pH of the HT group compared with the TN group.

The WHC was not influenced by the examined factors. However, the MFI increased in the 0.075% and 0.15% betaine treatments compared with the other groups. The percentage CL was highest (*p* < 0.01) for the control group breasts (Table 5). Additionally, the CL was higher (*p* < 0.05) in the TN group than the HT group. The lowest SF has observed in the breasts of birds fed 0.075% betaine, while the highest SF was obtained for the chickens that consumed 0.0% and 0.10% betaine (Table 5).

### 3.5. Blood Biochemical and Hematological Parameters

Table 6 shows the effect of the applied treatments on some serum biochemical parameters of broilers. Betaine supplementation changed (*p* < 0.05) only TGs and albumin concentrations. All serum biochemicals, except albumin, varied by temperature (*p* < 0.05), and all previous parameters were lower in the TN group than the HT group, except for glucose. The two-way interaction (Betaine × Temperature) was only significant (*p* < 0.05) for glucose concentration (Table 6). The results showed that betaine supplementation at 0.15% to the HT group increased the serum glucose level compared with the other groups.

However, the lowest glucose level was for the TN group’s unsupplemented control. Within betaine levels, HT increased the glucose level in the unsupplemented control and the 0.15% betaine-supplemented group. There were no changes in serum glucose within the other betaine levels due to temperature.

Table 7 shows the effect of the different treatments on the studied hematological parameters of the broilers. Heterophils, lymphocytes, and their ratio (H:L ratio) were altered (*p* < 0.001) by the HT treatment. The number of heterophils and the H:L ratio were lower in the TN group than the HT group, while the number of lymphocytes was higher in the HT group. There were no changes in the hematological blood characteristics due to the interaction between temperature and dietary betaine levels.

## 4. Discussion

Alleviating heat stress improves broiler performance, and determining the effectiveness of using dietary betaine supplementation to alleviate heat stress was the primary goal of the present trial. Through the first 18 days of age, in the absence of heat stress, dietary supplementation of betaine at 0.10% improved the PEF without affecting the FI, BWG, and FCR, which agrees with Uzunoğlu and Yalçin [16]. We found that 0.10% betaine supplementation was adequate for broiler chickens of 1–18 days of age. It should be mentioned that the experimental diets fed containing adequate nutrients, particularly methionine and choline, to avoid nutritional deficiency and declare the impact of betaine without, confound results with methyl donor agents (1).

It should be mentioned that, although several studies have been reported previously in the same topic, confirmation studies are needed in different parts of the world due to different enviromental conditions among various regions of the world. In our study, heat stress impaired growth performance and caused several changes in the metabolic and antioxidants status of the chickens, as also indicated for other poultry species [2,3,4,5,6]. It is known that the response to heat stress is influenced by exposure time, the temperature-humidity index, strain, and breed [4,7,8,10,32]. The current work exhibited reduced FI and impaired FCR in the HT group during the grower-finisher period, demonstrating that the birds did not adapt to temperatures that were above optimal. Heat stress is augmented by radiating temperature loss through the relocation of blood from the center to the periphery of the body, where it can be released to the environment [2,3,4,5,6,7]. This relocation of the blood during heat stress possibly causes hypoxia and tissue harm inside the gastrointestinal tract [22], influencing the digestive and absorptive system of the body and possibly compromising feed efficiency. Additionally, damage to the intestinal barrier can cause a gateway for pathogens, increasing the risk of disease [33,34].

One chief outcome of the current work was that betaine supplementation at 0.10% enhanced BWG, FCR, and PEF in broilers exposed to heat stress, suggesting that betaine may be a useful antistress nutritional tool. It should be noticed that betaine performs a fundamental function by controlling the cellular osmotic atmosphere during heat stress, reducing dryness by intensifying the WHC of the cell, and improving antioxidant balance [21,22,35]. Furthermore, betaine acts as a methyl group contributor, which is beneficial for the creation of protein in the body [1,17,23]. As an osmolyte, betaine lets proteins preserve their conformational steadiness in the existence of high uric acid levels and variations in cell salinity. Numerous tissues rely on betaine as an osmolyte, and it is found in the kidneys, brain, liver, intestines, and leukocytes [12].

Dietary betaine supplementation at three different concentrations (0.10%, 0.15%, and 0.20%) significantly improved FI, BWG, and FCR in heat-stressed broilers, especially at higher betaine levels [36]. Dietary betaine supplementation augmented BWG and enhanced FCR during 21–42 days of age, and they had no significant impact on the FI of broilers, as indicated by Shakeri et al. [22]. In growing rabbits, betaine enhanced BW and significantly boosted the FCR [37] in heat-stress environments. The incorporation of betaine in broiler feeds was efficient at advancing their performance [12,19]. However, Nutautaitė [17] reported that dietary betaine supplementation could not change BW and BWG, but it altered FCR in broilers. In contrast to our results, Uzunoğlu and Yalçin [16] showed that betaine dietary supplementation did not affect the growth performance indices of broilers, which could be due to different experimental conditions and the supplemented dose of betaine between the two studies. Different experimental results suggested the need for further experiments to escape the gap in contradictory results due to different levels of betaine used, animal species and environmental conditions, and composition of the experimental diets.

Our results showed that betaine supplementation did not affect broiler carcass traits, but heat stress decreased breast-meat percentage and increased fat percentages. It was imagined that dietary betaine would affect carcass weight and the relative weights of different carcass parts depending on its methyl group. This would boost methionine and cystine availability for protein synthesis [1,38] and glycine for protein creation. Similarly, it would contribute to decreasing fat precipitation in the body across numerous metabolic means [12]. However, this mode of action was not recorded herein, which agrees with Nutautaitė [17] and is contradictory to Nofal et al. [39]. In this regard, Attia et al. [32] stated that heat stress decreased the percentage of dressed carcasses, liver, and giblets. The same authors suggested that supplementing diets with 1 g betaine/kg caused complete retrieval of the adverse consequences. In contrast, El-Shinnawy [19] revealed that broilers that consumed betaine-supplemented diets had increased carcass and carcass part yield percentages.

In our study, the HT treatment decreased breast muscle colors L* and b*, CL, and pH after 24 h. Betaine supplementation changed the MFI, CL, and shearing force, but it did not affect the WHC and breast pH after 24 h. These findings contrast with those by Zhang et al. [40], who reported that breast meat from broilers raised under high temperature exhibited greater L* and CL than those reared under normal temperatures. The latter authors demonstrated that high temperatures could encourage rigor mortis, causing a quicker pH decline and higher L* value, leading to whitish, exudative meat traits [40]. Comparable results were also reported by Attia et al. [32], who indicated that heat stress and dietary betaine supplementation did not affect meat pH and color. In contrast, betaine addition at 0.5 g/kg diet lessened the WHC when compared with the control group, which could be due to increased water retention and osmolality of the cells. Nutautaitė et al. [17] showed that betaine dietary supplementation did not influence breast-muscle colors L*, b*, and the WHC in broilers. Additionally, broilers fed diets supplemented with 0.10% and 0.20% betaine had the highest CL, while birds consuming the control diet had the highest SF [17]. Additionally, Liu [41] found comparable findings to ours. In their trial, which aimed to reduce the impacts of transport stress on broilers, Chen et al. [24] reported that carcasses of broilers fed diets supplemented with betaine (0.05% or 0.10%) showed similar results for pH and CL compared with the control. Moreover, Al-Tamimi et al. [23] found that betaine addition did not alter the pH, CL, and shearing force of broiler carcass meat.

The escalation in blood albumin attributable to betaine incorporation was linked with its capability as a methyl group contributor, which is necessary for protein metabolism [1,36] and its role in improving immunity [16]. Similar outcomes were observed by Uzunoğlu and Yalçin [16], who stated that dietary betaine supplementation significantly reduced TGs without affecting the other blood parameters studied. Attia et al. [32] found that there was a decline in plasma glucose and total protein due to heat stress, although their results for plasma TGs were contradictory. Additionally, it was found that supplementing 1 g of betaine/kg diet resulted in complete relief in plasma glucose and incomplete relief in TGs and total protein [32]. Attia et al. [42] concluded that plasma glucose decreased in laying hens that were raised under heat-stress conditions and consumed a diet with no supplementation compared with their counterparts. Furthermore, El-Shinnawy [19] indicated that broilers fed diets supplemented with betaine had higher levels of TGs than those in the control group. In the present results, heat-stress impacted levels of glucose, TGs, cholesterol, and total protein. Similar findings were also reported by Ghasemi and Nari [42], who found significant differences in these biochemicals due to heat stress. They stated that dietary betaine supplementation did not impact the investigated blood biochemicals, except TGs [43].

Hematological parameters were negatively affected by the HT treatment, showing an increased number of heterophils, decreased number of lymphocytes, and an increased stress index (heterophil/lymphocyte ratio). However, there was no interaction between heat stress and betaine, which agrees with Abdelsattar et al. [44], who showed that betaine-supplemented diets in growing lambs receiving saline water did not cause a change in lymphocyte numbers.

The results of the heat-stress period (19–40 days of age) revealed that the heat challenge of 35 °C had a powerful effect on performance, meat characteristics, and blood parameters. Betaine fortification during days 19–40 of age during heat challenge reduced the negative impact on performance and improved production efficiency. This indicates that the supplementation of 0.10% betaine during the heat challenge was adequate for broilers. Further studies are required to test betaine’s ability, especially with different levels of methionine and choline, to reduce methionine supplementation and make betaine supplementation cost-effective.

Among the four tested levels of betaine, the practical level was found to be 0.1%, suggesting that betaine may be a useful nutritional tool under different stress conditions that deserve further investigation.

## 5. Conclusions

The best performance was achieved at 0.1% betaine fortifications with 84 g gain, 4.6 points improvement in FCR, and 24 points improvement in PEF compared to no betaine supplementation. The heat-stressed group consumed less feed (239 g), gained less weight (179 g), converted feed less efficiently (2.6 points), and, as a result, had lower FEF (29 points) as compared to the TN group. Conclusively, heat challenge had a powerful effect on growth performance, meat characteristics, and blood parameters, especially during the grower-finisher period. Betaine fortification (0.1%) during heat challenge reduced the negative impact on performance and improved production efficiency. As an antistress agent, the role of betaine should be addressed in further research and explore its application benefits.

## Figures and Tables

**Table 1:** Dietary ingredients and chemical composition of starter (1–18 days of age) and finisher (19–40 days of age) diets.

	1–18 d		19–40 d
Ingredients	Basal Starter		Basel Finisher
		%	
Corn	59.105		62.00
Soybean meal	27.15		25.35
Corn gluten meal	6.70		4.275
Corn oil	2.53		3.72
Dicalcium phosphate	2.20		2.03
Limestone	0.70		0.57
Salt	0.40		0.40
VM Mix ^1^	0.50		0.50
DL-Methionine	0.20		0.76
Lysine-HCL	0.33		0.24
Threonine	0.135		0.105
Choline chloride	0.05		0.05
Total	100		100
Calculated ^1^ and determined ^2^ composition			
ME, kcal/kg ^1^	3050		3150
Crude protein, % ^2^	22.0		21.3
Crude fat,% ^2^	35.3		34.6
Crude fiber,% ^2^	35.5		46.2
Crude ash,% ^2^	60.3		54.2
Lysine, % ^1^	1.22		1.10
Sulfur amino acids, % ^2^	0.89		0.80
Threonine, % ^2^	0.80		0.72
Calcium, % ^2^	0.94		0.85
Phosphorus, % ^2^	0.45		0.42

^1^ Vitamin–mineral premix contains the following per kg: vitamin A, 2,400,000 IU; vitamin D, 1,000,000 IU; vitamin E, 16,000 IU; vitamin K, 800 mg; vitamin B1, 600 mg; vitamin B_2_, 1600 mg; vitamin B_6_, 1000 mg; vitamin B_12_, 6 mg; niacin, 8000 mg; folic acid, 400 mg; pantothenic acid, 3000 mg; biotin 40 mg; antioxidant, 3000 mg; cobalt, 80 mg; copper, 2000 mg; iodine, 400; iron, 1200 mg; manganese, 18,000 mg; selenium, 60 mg, and zinc, 14,000 mg; 25% lysine; 32% methionine; ^2^ Determined values.

**Table 2:** Cumulative feed intake (FI), body weight gain (BWG), feed conversion ratio (FCR), and production efficiency factor (PEF) of broiler chickens given experimental diets at 0–18 days of age.

		Performance			
Treatment	Betaine Added	FI (g)	BWG (g)	FCR (g:g)	PEF
1	0.0%	761	577	1.320	267 ^b^
2	0.075%	761	581	1.311	270 ^b^
3	0.10%	772	601	1.286	285 ^a^
4	0.15%	748	576	1.299	271 ^b^
SEM		8.96	8.21	0.009	4.92
*p* value		NS	NS	NS	*

^a,b^ Means within a column within the same factor with different superscript letters differ significantly (* *p* < 0.05, NS: not significant). NS, not significant; SEM, standard error of the mean.

**Table 3:** The cumulative effect of betaine fortifications and a heat challenge of 35 °C on feed intake (FI), live weight gain (BWG), feed conversion ratio (FCR), and production efficiency factor (PEF) of broiler chickens during 19–40 days of age.

			Performance			
Treatment	Betaine	Temperature	FI (g)	BWG (g)	FCR (g:g)	PEF
Interaction between betaine and temperature						
1	0.0%	TN	2981	1851	1.611	373
2	0.0%	HT	2704	1658	1.635	346
3	0.075%	TN	2887	1853	1.559	384
4	0.075%	HT	2706	1705	1.589	363
5	0.10%	TN	3004	1917	1.570	397
6	0.10%	HT	2781	1758	1.583	370
7	0.15%	TN	2972	1906	1.561	393
8	0.15%	HT	2697	1690	1.597	352
SEM±			35.84	30.05	0.014	7.76
Betaine average						
0.0%			2843	1754 ^b^	1.623 ^a^	359 ^b^
0.075%			2797	1779 ^ab^	1.574 ^b^	374 ^ab^
0.10%			2893	1838 ^a^	1.577 ^b^	383 ^a^
0.15%			2834	1798 ^ab^	1.579 ^b^	372 ^ab^
Temperature average						
Temperature average						
TN			2961 ^a^	1882 ^a^	1.575 ^a^	387 ^a^
HT			2722 ^b^	1703 ^b^	1.601 ^b^	358 ^b^
Statistical probabilities						
Betaine			NS	*	**	*
Temperature			***	***	*	***
Betaine × Temperature			NS	NS	NS	NS

^a,b^ Means within a column within the same factor with different superscripts differ significantly (* *p* < 0.05, ** *p* < 0.01, *** *p* < 0.001; NS, not significant). SEM: standard error of the mean.

**Table 4:** Effect of betaine fortifications and temperature on carcass part yields as percentages of broiler dressed weight of 40-day-old broiler chickens.

			Dressing	Breast	Leg	Fat	Liver	IRW
Treatment	Betaine	Temperature	(%)	(%)	(%)	(%)	(%)	(%)
Interaction between betaine and temperature								
1	0.0%	TN	72.5	39.5	29.9	1.7	2.3	3.8 ^a^
2	0.0%	HT	72.9	38.4	30.5	2.3	2.2	3.5 ^ab^
3	0.075%	TN	72.9	39.5	30.2	1.7	2.2	3.6 ^ab^
4	0.075%	HT	72.9	38.5	30.1	1.9	2.3	3.7 ^a^
5	0.10%	TN	73.5	40.0	28.9	1.8	2.3	3.4 ^b^
6	0.10%	HT	72.9	38.0	30.8	1.7	2.1	3.8 ^a^
7	0.15%	TN	73.1	40.4	29.8	1.7	2.2	3.6 ^ab^
8	0.15%	HT	73.3	40.1	29.6	2.0	2.0	3.5 ^ab^
SEM±			0.38	39.5	0.52	0.16	0.10	0.11
Betaine average								
0.0%			72.7	38.9	30.2	2.0	2.3	3.6
0.075%			73.0	39.0	30.1	1.8	2.2	3.7
0.10%			73.2	39.0	29.8	1.7	2.2	3.6
0.15%			73.2	40.3	29.7	1.8	2.1	3.5
Temperature average								
TN			73.0	39.9 ^a^	29.7	1.7 ^b^	2.2	3.6
HT			73.0	38.7 ^b^	30.3	2.0 ^a^	2.1	3.6
Statistical probabilities								
Betaine			NS	NS	NS	NS	NS	NS
Temperature			NS	**	NS	*	NS	NS
Betaine × Temperature			NS	NS	NS	NS	NS	*

^a,b^ Means within a column within the same factor with different superscripts differ significantly (* *p* < 0.05, ** *p* < 0.01; NS, not significant). SEM: standard error of the mean.

**Table 5:** Effect of betaine fortifications and temperature on meat parameters of 40-day-old broiler chickens.

			pH-10 min.	Temp.-10 min. (°C)	Color-10 min.			pH-24 h	Color-24 h			WHC	MFI	CL	SF (Kgf/g)
Treatment	Betaine	Temperature			L*	a*	b*		L*	a*	b*				
Interaction between betaine and temperature															
1	0.0%	TN	6.60	31.9 ^b^	47.5	3.64 ^b^	8.67	6.44 ^a^	50.9	3.83	9.13	0.24	62.1	39.7	1.57
2	0.0%	HT	6.48	29.2	44.7	4.44 ^ab^	7.90	6.34 ^c^	47.1	4.60	10.31	0.23	57.2	35.7	1.81
3	0.075%	TN	6.56	31.8 ^b^	45.4	4.02 ^ab^	8.87	6.37 ^bc^	48.8	4.22	11.10	0.22	63.9	35.1	1.28
4	0.075%	HT	6.52	30.5 ^c^	45.9	3.24 ^b^	8.54	6.38 ^bc^	49.1	3.78	10.32	0.19	65.0	30.6	1.50
5	0.10%	TN	6.61	32.2 ^b^	48.8	4.09 ^ab^	8.30	6.40 ^ab^	49.4	4.06	10.55	0.23	58.8	30.7	1.99
6	0.10%	HT	6.45	33.1 ^a^	46.2	3.69 ^ab^	7.16	6.35 ^c^	47.3	5.00	9.48	0.22	52.9	31.3	1.72
7	0.15%	TN	6.50	31.7 ^b^	47.8	3.14 ^b^	9.71	6.35 ^c^	48.3	3.92	11.59	0.23	62.3	33.9	1.63
8	0.15%	HT	6.49	32.9 ^a^	45.4	4.97 ^a^	8.25	6.38 ^bc^	46.2	5.13	8.77	0.21	63.6	28.7	1.54
SEM±			0.03	0.18	1.11	0.46	0.55	0.018	1.23	0.47	0.075	0.014	2.31	1.64	0.13
Betaine average															
0.0%			6.54	30.6 ^c^	46.1	4.03	8.29	6.39	49.0	4.21	9.72	0.24	59.7 ^bc^	36.2 ^a^	1.69 ^ab^
0.075%			6.54	31.1 ^b^	45.7	3.63	8.71	6.37	48.9	3.99	10.71	0.21	64.5 ^a^	32.8 ^b^	1.39 ^c^
0.10%			6.53	32.6 ^a^	47.5	3.89	7.74	6.38	48.4	4.53	10.01	0.22	55.8 ^c^	31.0 ^b^	1.86 ^a^
0.15%			6.49	32.3 ^a^	46.6	4.06	8.98	6.36	47.2	4.53	10.18	0.22	62.9 ^ab^	31.3 ^b^	1.59 ^bc^
Temperature average															
	TN		6.57 ^a^	31.9 ^a^	47.4 ^a^	3.72	8.89 ^a^	6.39 ^a^	49.3 ^a^	4.00	10.59	0.23	61.8	34.1 ^a^	1.62
	HT		6.49 ^b^	31.4 ^b^	45.5 ^b^	4.08	7.97 ^b^	6.36 ^b^	47.4 ^b^	4.62	9.72	0.22	59.7	31.6 ^b^	1.64
Statistical probabilities															
Betaine			NS	***	NS	NS	NS	NS	NS	NS	NS	NS	**	**	**
Temperature			**	***	*	NS	*	*	*	NS	NS	NS	NS	*	NS
Betaine × Temperature			NS	***	NS	*	NS	**	NS	NS	NS	NS	NS	NS	NS

pH-10, Temp.-10, and color-10 min = pH, temperature, and color measurements of the breast 10 min postmortem, respectively; pH-24 h, Temp.-24 h, and color-24 h = pH, temperature, and color measurements of the breast 24 h post mortem, respectively; CL, cooking loss; WHC, water-holding capacity; MFI, myofibril fragmentation index; SF, shearing force. ^abc^ The means within a coulumn within the same factor with different superscripts differ significantly (* *p* < 0.05, ** *p* < 0.01, *** *p* < 0.001; NS, not significant). SEM: standard error of the mean.

**Table 6:** Effect of betaine fortifications and temperature on pooled blood parameters of 35-day-old broiler chickens.

			Glucose	TGs ^1^	Cholesterol	TP ^2^	Albumin	Globulin	A:G ^3^
Treatment	Betaine	Temperature	(mg/dL)	(mg/dL)	(mg/dL)	(g/dL)	(g/dL)	(g/dL)	
Interaction between betaine and temperature									
1	0.0%	TN	191.0 ^c^	78.9	95.0	2.9	1.7	1.2	1.6
2	0.0%	HT	218.4 ^b^	71.2	102.0	3.1	1.8	1.3	1.6
3	0.075%	TN	210.2 ^bc^	72.6	89.5	2.7	1.7	1.0	1.9
4	0.075%	HT	221.6 ^b^	63.2	92.4	3.1	1.5	1.6	1.1
5	0.10%	TN	202.6 ^bc^	68.2	90.6	3.0	1.7	1.3	1.5
6	0.10%	HT	198.7 ^bc^	64.2	95.8	3.1	1.8	1.3	1.4
7	0.15%	TN	198.7 ^bc^	73.3	92.0	3.0	1.7	1.3	1.5
8	0.15%	HT	242.9 ^a^	63.3	95.5	2.9	1.5	1.5	1.2
SEM±			8.3	3.33	3.02	0.09	0.08	0.11	0.19
Betaine average									
0.0%			204.7	75.0 ^a^	98.5	3.0	1.8 ^a^	1.2	1.6
0.075%			215.9	67.9 ^b^	91.0	2.9	1.6 ^ab^	1.3	1.5
0.10%			200.6	66.2 ^b^	93.2	3.1	1.7 ^ab^	1.3	1.5
0.15%			220.8	68.3 ^b^	93.8	2.9	1.6 ^b^	1.4	1.3
Temperature average									
TN			200.6 ^b^	73.3 ^a^	91.8 ^b^	2.9 ^b^	1.7	1.2 ^b^	1.6 ^a^
HT			220.4 ^a^	65.5 ^b^	96.0 ^a^	3.1 ^a^	1.6	1.4 ^a^	1.3 ^b^
Statistical probabilities									
Betaine			NS	*	NS	NS	*	NS	NS
Temperature			**	**	*	*	NS	**	*
Betaine × Temperature			*	NS	NS	NS	NS	NS	NS

^a,b,c^ Means within a column within the same factor with different superscripts differ significantly (* *p* < 0.05, ** *p* < 0.01; NS, not significant). SEM, standard error of the mean; ^1^ TGs, triglycerides, ^2^ TP, total protein, ^3^ A:G ratio, albumin to globulin ratio.

**Table 7:** Effect of betaine fortifications and temperature on pooled blood parameters of 35-day-old broiler chickens.

			Hetophile	Lymphocyte	H:L ^1^
Treatment	Betaine	Temperature	(%)	(%)	Ratio
Interaction between Betaine and temperature					
1	0.0%	TN	12.0	88.0	0.14
2	0.0%	HT	27.0	71.0	0.42
3	0.075%	TN	14.0	86.0	0.16
4	0.075%	HT	29.0	70.0	0.44
5	0.10%	TN	15.0	85.0	0.18
6	0.10%	HT	28.0	71.0	0.42
7	0.15%	TN	12.0	87.0	0.14
8	0.15%	HT	25.0	73.0	0.36
SEM±			2.10	2.33	0.05
Betaine average					
0.0%			20.0	79.0	0.28
0.075%			21.0	78.0	0.30
0.10%			21.0	78.0	0.30
0.15%			19.0	80.0	0.25
Temperature average					
TN			13.0 ^b^	86.0 ^a^	0.16 ^b^
HT			27.0 ^a^	71.0 ^b^	0.41 ^a^
Statistical probabilities					
Betaine			NS	NS	NS
Temperature			***	***	***
Betaine × Temperature			NS	NS	NS

^a,b^ The means within a column within the same factor with different superscripts differ significantly (*** *p* < 0.001; NS, not significant). SEM, standard error of the mean; ^1^ H:L ratio, heterophil to lymphocyte ratio.

## Data Availability

All data are presented in the text and tables of this manuscript.

## References

[1] Attia Y.A., Hsaan R.A., Shehatta M.H., Abd El-Hady S. (2005). Growth, carcass quality and serum constituents of slow growing chicks as affected by betaine addition to diets containing 2 different levels of methionine. Int. J. Poult. Sci..

[2] Attia Y.A., Hassan S.S. (2017). Broiler tolerance to heat stress at various dietary protein/energy levels. Eur. Poult. Sci..

[3] Attia Y.A., Al-Harthi M.A., Elnaggar A.S. (2018). Productive, physiological and immunological responses of two broiler strains fed different dietary regimens and exposed to heat stress. Ital. J. Anim. Sci..

[4] Farghly M.F.A., Mahrose K.M., Galal A.E., Ali R.M., Ahmad E.A.M., Rehman Z.U., Ullah Z., Ding C. (2018). Implementation of different feed withdrawal times and water temperatures in managing turkeys during heat stress. Poult. Sci..

[5] Farghly M.F.A., Mahrose K.H.M., Mahmoud G.B., Ali R.M., Daghash W., Metwally K.A., Abougaba M.S. (2020). Lighting programs as an appliance to improve growing New Zealand white rabbit’s performance. Int. J. Biometeorol..

[6] Farghly M.F.A., Mahrose K.M., Ullah Z., Rehman Z.U., Ding C. (2017). Influence of swimming time in alleviating the deleterious effects of hot summer on growing Muscovy duck performance. Poult. Sci..

[7] Rizk Y.S., Fahim H.N., Beshara M.M., Mahrose K.M., Awad A.L. (2019). Response of duck breeders to dietary L-Carnitine supplementation during summer season. An. Acad. Bras. Ciências.

[8] Awad A., Fahim H., El-Shhat A.E., Mahrose K.H., Shazly S. (2021). Dietary *Echinacea purpurea* administration enhanced egg laying performance, serum lipid profile, antioxidant status and semen quality in duck breeders during summer season. J. Anim. Physiol. Anim. Nutr..

[9] De Silva P., Kalubowila A. (2012). Influence of feed withdrawal for three hour time period on growth performance and carcass parameters of later stage of male broiler chickens. Iran. J. Appl. Anim. Sci..

[10] Hassan F.A., Mahrose K.M., Basyony M.M. (2016). Effects of grape seed extract as a natural antioxidant on growth performance, carcass characteristics and antioxidant status of rabbits during heat stress. Arch. Anim. Nutr..

[11] Abd El-Hack M.E., Mahrose K.H.M., Arif M., Chaudhry M.T., Saadeldin I.M., Saeed M., Soomro R.N., Abbasi I.H.R., Rehman Z.U. (2017). Alleviating the environmental heat burden on laying hens by feeding on diets enriched with certain antioxidants (vitamin E and selenium) individually or combined. Environ. Sci. Pollut. Res..

[12] Sakomura N.K., Barbosa N.A.A., Longo F.A., da Silva E.P., Bonato M.A., Fernandes J.B.K. (2013). Effect of dietary betaine supplementation on the performance, carcass yield, and intestinal morphometrics of broilers submitted to heat stress. Braz. J. Poult. Sci..

[13] Ratriyanto A., Indreswari R., Nuhriawangsa A.M.P. (2017). Effects of dietary protein level and betaine supplementation on nutrient digestibility and performance of Japanese quails. Braz. J. Poult. Sci..

[14] Attia Y.A., El-Hamid A.E.A., Abdallah A.A., Berika M.A., El-Gandy M.F., Sahin K., Abou-Shehema B.M. (2018). Effect of betaine, vitamin C, and vitamin E on egg quality, hatchability, and markers of liver and renal functions in dual-purpose breeding hens exposed to chronic heat stress. Eur. Poult. Sci..

[15] Attia Y.A., El-Naggar A.S., Abou-Shehema B.M., Abdella A.A. (2019). Effect of Supplementation with Trimethylglycine (Betaine) and/or Vitamins on Semen Quality, Fertility, Antioxidant Status, DNA Repair and Welfare of Roosters Exposed to Chronic Heat Stress. Animals.

[16] Uzunoğlu K., Yalçin S. (2019). Effects of dietary supplementation of betaine and sepiolite on performance and intestinal health in broilers. Ank. Üniv. Vet. Fak. Derg..

[17] Nutautaitė M., Alijošius S., Bliznikas S., Šašytė V., Vilienė V., Pockevičius A., Racevičiūtė-Stupelienė A. (2020). Effect of betaine, a methyl group donor, on broiler chicken growth performance, breast muscle quality characteristics, oxidative status and amino acid content. Ital. J. Anim. Sci..

[18] Summers J.D. (2013). Effect of Choline or Betaine Supplementation on Broilers Exposed to Different Temperature Treatments. Master’s Thesis.

[19] El-Shinnawy A.M. (2015). Effect of betaine supplementation to methionine adequate diet on growth performance, carcass characteristics, some blood parameters and economic efficiency of broilers. J. Anim. Poult. Prod..

[20] Willingham B.D., Ragland T.J., Ormsbee M.J. (2020). Betaine supplementation may improve heat tolerance: Potential mechanisms in humans. Nutrients.

[21] Wasti S., Sah N., Mishra B. (2020). Impact of heat stress on poultry health and performances, and potential mitigation strategies. Animals.

[22] Shakeri M., Cottrell J.J., Wilkinson S., Ringuet M., Furness J.B., Dunshea F.R. (2018). Betaine and antioxidants improve growth performance, breast muscle development and ameliorate thermoregulatory responses to cyclic heat exposure in broiler chickens. Animals.

[23] Al-Tamimi H., Mahmoud K., Al-Dawood A., Nusairat B., Bani Khalaf H. (2019). Thermotolerance of broiler chicks ingesting dietary betaine and/or creatine. Animals.

[24] Chen R., When C., Gu Y., Wang C., Chen Y., Zhuang S., Zhou Y. (2020). Dietary betaine supplementation improves meat quality of transported broilers through altering muscle anaerobic glycolysis and antioxidant capacity. J. Sci. Food Agric..

[25] Commission International de l’Eclairage (1977). Official Recommendations on Uniform Colour Space, Colour Difference Equations and Metric Colour Terms. Color Res. Appl..

[26] Culler R.D., Parrish F.C., Smith G.C., Cross H.R. (1978). Relationship of myofibril fragmentation index to certain chemical, physical and sensory characteristics of bovine longissimus muscle. J. Food Sci..

[27] Hamm R. (1960). Biochemistry of meat hydration. Adv. Food Res..

[28] Wilhelm A.E., Maganhini M.B., Hernández-Blazquez F.J., Ida E.I., Shimokomaki M. (2010). Protease activity and the ultrastructure of broiler chicken PSE (pale, soft, exudative) meat. Food Chem..

[29] Attia Y.A., Al-Harthi M.A., Korish M.M., Shiboob M.M. (2016). Evaluation of the broiler meat quality in the retail market: Effects of type and source of carcasses. Rev. Mex. Cienc. Pecu..

[30] Wheeler T.L., Cundiff L.V., Shackelford S.D., Koohmaraie M. (2005). Characterization of biological types of cattle (Cycle VII): Carcass, yield, and longissimus palatability traits. J. Anim. Sci..

[31] SAS Institute (2004). SAS/SRATI User’s Guide, Statistics.

[32] Attia Y.A., Hassan R.A., Qota E.M. (2009). Recovery from adverse effects of heat stress on slow-growing chicks in the tropics 1: Effect of ascorbic acid and different levels of betaine. Trop. Anim. Health Prod..

[33] Keita Å.V., Söderholm J.D. (2010). The intestinal barrier and its regulation by neuroimmune factors. Neurogastroenterol. Motil..

[34] Dunshea F.R., Leury B.J., Fahri F.T., Digiacomo K., Hung A., Chauhan S.S., Clarke I.J., Collier R.J., Little S., Baumgard L.H. (2013). Amelioration of thermal stress impacts in dairy cows. Anim. Prod. Sci..

[35] Ratriyanto A., Mosenthin R. (2018). Osmoregulatory function of betaine in alleviating heat stress in poultry. J. Anim. Physiol. Anim. Nutr..

[36] Chand N., Naz S., Maris H., Khan R.U., Khan S., Qureshi M.S. (2017). Effect of betaine supplementation on the performance and immune response of heat stressed broilers. Pak. J. Zool..

[37] Hassan R.A., Ebeid T.A., Abd El-Lateif A.I., Ismail N.B. (2011). Effect of dietary betaine supplementation on growth, carcass and immunity of New Zealand White rabbits under high ambient temperature. Livest. Sci..

[38] Mcdevitt R.M., Mack S., Wallis I.R. (2000). Can betaine partially replace or enhance the effect of methionine by improving broiler growth and carcass characteristics. Br. Poult. Sci..

[39] Nofal M.E., Magda A., Galal S.M.M., Mousa M., Doaa M.M., Yassein M., Bealsh A.M.A. (2015). Effect of dietary betaine supplementation on productive, physiological and immunological performance and carcass characteristic of growing developed chicks under the condition of heat stress. Egypt. Poult. Sci. J..

[40] Zhang Z.Y., Jia G.Q., Zuo J.J., Zhang Y., Lei J., Ren L., Feng D.Y. (2012). Effects of constant and cyclic heat stress on muscle metabolism and meat quality of broiler breast fillet and thigh meat. Poult. Sci..

[41] Liu W., Yuan Y., Sun C., Balasubramanian B., Zhao Z., An L. (2019). Effects of dietary betaine on growth performance, digestive function, carcass traits, and meat quality in indigenous yellow-feathered broiler under long-term heat stress. Animals.

[42] Attia Y.A., El-Hamid A.E.-H.E.A., Abedalla A.A., Berika M.A., Al-Harthi M.A., Kucuk O., Sahin K., Abou-Shehema B.M. (2016). Laying performance, digestibility and plasma hormones in laying hens exposed to chronic heat stress as affected by betaine, vitamin C, and/or vitamin E supplementation. Springerplus.

[43] Ghasemi H.A., Nari N. (2020). Effect of supplementary betaine on growth performance, blood biochemical profile, and immune response in heat-stressed broilers fed different dietary protein levels. J. Appl. Poult. Res..

[44] Abdelsattar M.M., Hussein A.M.A., Haridy M., Abd El-Ati M.N., Saleem A.M., Zhang N. (2019). Betaine counteracts the harmful effects of saline water induced to growing lambs. Egypt. J. Sheep Goat Sci..

